# Scleritis and associated systemic diseases: contribution of systemic examination, follow-up, and additional investigations

**DOI:** 10.1186/s12348-025-00566-7

**Published:** 2025-12-25

**Authors:** Mathilde Soubrier, Caroline Vasseneix, Robin Jacquot, Arthur Bert, Mathieu Gerfaud-Valentin, Thibaud Mathis, Laurent Kodjikian, Pascal Sève

**Affiliations:** 1 Département d’ophtalmologie, Hôpital Universitaire Edouard Herriot, Hospices Civils de Lyon, Lyon, France; 2 Département d’ophtalmologie, Hôpital Universitaire de la Croix-Rousse, Hospices Civils de Lyon, Lyon, France; 3 Département de médecine interne, Hôpital Universitaire de la Croix-Rousse, Hospices Civils de Lyon, Lyon, France; 4 Research on Healthcare Performance (RESHAPE), INSERM U1290, Université Claude Bernard Lyon 1, Lyon, France; 5 UMR5510 MATEIS, CNRS, INSA Lyon, Université Claude Bernard Lyon 1, Villeurbanne, France

**Keywords:** Anti-neutrophil cytoplasmic antibodies (ANCA), Associated disease, Granulomatosis with polyangiitis (GPA), Rheumatoid factor, Scleritis

## Abstract

**Background:**

Identifying underlying disease associations in patients with scleritis remains a clinical challenge. This study aimed to assess the contribution of systemic examination, longitudinal follow-up, and additional investigations to the identification of associated diseases in patients with scleritis.

**Methods:**

We retrospectively reviewed 98 patients with scleritis in whom no associated disease had been identified at presentation, assessed in two internal medicine departments in Lyon between July 2011 and September 2023. Data were collected at the initial presentation, prior to the identification of any underlying disease. The primary outcome was the contribution of systemic examination to the identification of associated diseases.

**Results:**

After a median follow-up of 42 months [15.5–100], an associated disease was identified in 37 patients (37.8%), including 23 systemic diseases (23.5%), 12 infectious diseases (12.2%), and 2 cases of drug-induced scleritis (2.0%). Systemic examination contributed to the identification of an associated disease in 20 patients (20.4%), mainly through ear, nose and throat (ENT) and dermatological assessments. In 70% of these patients, systemic manifestations preceded the first episode of scleritis, while in the remaining 30% they developed during follow-up. In three patients, scleritis was the initial and sole manifestation, and the diagnosis was established through additional investigations during follow-up. The positive predictive value (PPV) of non-specific anti-neutrophil cytoplasmic antibodies (ANCA) was 29%, with a negative predictive value (NPV) of 98%. When specific ANCA testing was performed, the PPV increased to 86%. Repeating laboratory investigations during follow-up did not yield additional diagnoses.

**Conclusion:**

Systemic examination contributed to the identification of an associated disease in one-fifth of patients. Scleritis may be the first clinical manifestation of an underlying systemic disease, supporting the need for systematic and prolonged follow-up. These findings also underline the diagnostic value of specific ANCA testing, whereas repeating laboratory investigations after an initial negative assessment appears of limited utility.

**Supplementary Information:**

The online version contains supplementary material available at 10.1186/s12348-025-00566-7.

## Background

Identifying underlying disease associations in patients with scleritis remains a clinical challenge. This study aimed to assess the contribution of systemic examination, longitudinal follow-up, and additional investigations to the identification of associated diseases in patients with scleritis.

## Introduction

Scleritis is an uncommon and typically painful inflammation of the sclera that can lead to vision loss. Globally, the reported prevalence of scleritis varies considerably, ranging from 1.0 per 100,000 person-years in Australia (2014) [1] to 93.6 per 100,000 in the United Kingdom (2018) [2], reflecting geographical and demographic differences.

The systemic associations of scleritis vary among patients; an underlying systemic disease is identified in approximately one-third of cases. Common associated diseases include granulomatosis with polyangiitis (GPA), rheumatoid arthritis (RA), spondyloarthritis, relapsing polychondritis (RP), and systemic lupus erythematosus (SLE) [3–5]. Scleritis can also be secondary to infectious diseases in approximately 10% of cases, including tuberculosis, syphilis, and herpes viruses [6–9]. More rarely, scleritis may be the result of adverse drug reactions or ocular surgery [10, 11]. However, in the majority of cases, scleritis remains idiopathic despite the initial assessment [3, 4, 12, 13]. Ophthalmological examination, including the location of scleritis according to Watson and Hayreh’s classification [14], offers limited insight into identifying associated systemic diseases. Nevertheless, the aetiology influences patient outcomes; for instance, necrotising scleritis is more frequently associated with systemic diseases [15].

A systemic examination is therefore required to identify systemic manifestations that may point toward an underlying systemic disease. This examination is usually performed by a specialist, most often an internist. Despite its central role in clinical practice, the actual contribution of systemic clinical examination has never been formally evaluated, representing a significant knowledge gap.

To date, no recommendation exists regarding the optimal clinical, laboratory, or imaging strategy for the assessment of scleritis.

While some laboratory tests—such as ANCA (by immunofluorescence) and rheumatoid factor (RF)—have been suggested for initial evaluation [12], the diagnostic performance of ANCA, the added value of specific ANCA testing, and the usefulness of repeating these tests during follow-up remain unknown.

Although a few studies have investigated the temporal relationship between scleritis and systemic disease [3, 16, 17], none has specifically characterised the clinical features that may help identify an associated systemic disease.

We focused primarily on patients with scleritis without a previously identified disease and hypothesised that a comprehensive systemic assessment, combined with longitudinal follow-up and targeted additional investigations, could improve the detection of associated systemic diseases.

The aim of this study was therefore to evaluate the contribution of systemic clinical examination, longitudinal follow-up, and laboratory and imaging investigations in identifying systemic associations in patients with scleritis.

## Patients and methods

### Study design and population

This observational and retrospective cohort was conducted in two centres of the *Hospices Civils de Lyon* (HCL; *Croix-Rousse University Hospital* and *Edouard Herriot University Hospital*, Lyon, France). Patients were identified using the HCL software by searching the keyword “scleritis”. We included all adult patients diagnosed with scleritis and referred to the internal medicine department of at least one centre between July 2011 and September 2023 for systemic evaluation. These departments are specialised in systemic inflammatory diseases, excluding inflammatory rheumatic disorders (such as RA, spondyloarthritis), which are managed in dedicated rheumatology units.

The diagnosis of scleritis had to be confirmed by an ophthalmologist. Patients with a previously identified associated systemic disease were excluded. Patients were also excluded if they had been seen fewer than two times across the participating hospitals or if the diagnosis of scleritis was uncertain.

According to French law (no. 2004 − 806, 9 August 2004), and because the data were collected retrospectively without modification of patient management, this study did not require research ethics committee approval. However, the cohort study received approval from the local ethics committee (Hospices Civils de Lyon, IRB n° 00013204) in September 2024 (n° 24-5076).

### Clinical data

For data collection, patients’ medical records were reviewed. Ophthalmological data were collected and consisted of the features and location of scleritis, as well as associated ophthalmological manifestations, such as uveitis and glaucoma. Anterior scleritis was defined as diffuse, nodular, and necrotising with or without inflammation according to the classification of Watson and Hayreh [14].

The diagnosis of posterior scleritis was established using B-mode ultrasound or magnetic resonance imaging (MRI) (T1-weighted sequences with fat suppression after gadolinium injection, and T2-weighted or STIR sequences), which revealed scleral thickening, periscleral oedema and sometimes the presence of a “T-sign”. The “T-sign” corresponds to the accumulation of fluid behind the sclera and the optic nerve, although it is inconsistently found [18–20]. Pan-scleritis was defined as combined anterior and posterior scleritis.

Uveitis associated with scleritis was defined as the presence of at least five cells in the anterior chamber on slit-lamp examination. We determined whether uveitis preceded the onset of scleritis, occurred simultaneously, or developed during the follow-up. Intraocular hypertension was defined as an intraocular pressure greater than 21 mmHg [21]. Associated glaucoma was considered when there was evidence of visual field loss and thinning of the peripapillary retinal nerve fibre layer [21].

The data collected from ophthalmological medical records included sex, age at scleritis diagnosis, laterality of scleritis, type of scleritis, and ocular complications.

The data collected from internal medicine medical records included the patient’s medical history at the first presentation, the presence of clinical manifestations suggestive of a systemic disease, the follow-up duration, the course of these manifestations, as well as any new systemic diseases identified during or after the episode of scleritis and the time to diagnosis.

### Laboratory, imaging, and pathology tests

Additional data collected included results of laboratory, imaging, and pathology investigations. Laboratory testing was systematic but not standardised. We collected results of: complete blood count (CBC), C-reactive protein (CRP), serum protein electrophoresis (SPE), angiotensin-converting enzyme (ACE; positivity threshold > 70 IU/L), syphilis serology (TPHA-VDRL or antigen test), QuantiFERON^®^-TB Gold, ANCA by indirect immunofluorescence (cytoplasmic or perinuclear fluorescence) and enzyme-linked immunosorbent assay (ELISA; positivity threshold > 3 IU/mL for anti-myeloperoxidase [anti-MPO] or anti-proteinase 3 [anti-PR3]), rheumatoid factor (RF; positivity threshold > 20 IU/mL), anti-cyclic citrullinated peptide (anti-CCP; positivity threshold > 3 IU/mL), anti-nuclear antibodies (ANA; positivity threshold > 1/80), and Human Leukocyte Antigen (HLA) typing. The results of the following imaging were also collected: chest X-ray, chest computed tomography (CT) scan, sinus CT scan, osteoarticular imaging (bone X-rays and MRI), and positron emission tomography scan (PET scan). For each modality, the device models, acquisition protocols, and calibration procedures used in the participating hospitals were retrieved when available and are provided in the Supplementary Material. We also collected the results of specialist examinations (ear, nose, and throat [ENT] specialist, gastroenterology, rheumatology, and dermatology), as well as pathology results (nasal and sinus biopsies, endobronchial biopsy of the carina, salivary gland biopsy, conjunctival biopsy).

Briefly, the diagnostic criteria used were: the American College of Rheumatology (ACR) and the European Alliance of Associations for Rheumatology (EULAR) 2022 criteria for ANCA-associated vasculitis [22]; the ACR/EULAR 2010 criteria for RA [23]; the international study group for Behcet’s disease [24]; the Assessment of SpondyloArthritis International Society (ASAS) for spondyloarthritis [25]; the Michet criteria for RP [26]; the ACR 2016 criteria for Sjögren’s syndrome [27]; the Montreal criteria for Crohn’s disease [28]; and the International Workshop on Sarcoidosis (IWOS) criteria [29], as well as an update of the international guidelines for sarcoidosis [30]. The identification of herpetic scleritis was based on unilateral scleritis, sometimes with suggestive corneal anaesthesia and/or round-shaped iris atrophy, responding to treatment with valaciclovir and, when necessary, an aqueous humour tap [7]. Tuberculosis-related scleritis was identified when the scleritis was resistant to first-line therapy, with a positive QuantiFERON^®^-TB Gold test, clinical improvement under anti-tuberculous therapy, and without other aetiology of scleritis after a follow-up of at least 24 months [31, 32].

### Statistical analysis

Continuous variables were expressed as mean ± standard deviation (SD) or medians [interquartile range (IQR)]. The assumption of normality was assessed using the Shapiro-Wilk test. Categorical variables were expressed as counts (percentages). Categorical variables were compared using Fisher’s exact test and continuous variables using Kruskal-Wallis test. When omnibus p-value was less than 0.05, post-hoc test for two by two multiple comparisons was performed: Dunn’s test following Kruskal-Wallis and Marascuilo’s procedure following Fisher’s exact test. Risk factors for the development of systemic disease (GPA) among patients with idiopathic scleritis after initial presentation were also analyzed using the Fisher’s exact test: odds ratios (OR), likelihood ratios, sensitivity, specificity, as well as negative and positive predictive values were determined. Statistical analyses were performed using Stata (version 15). Differences were considered statistically significant at *p* < 0.05. No a priori sample size calculation was performed, as this was an exploratory retrospective study including all eligible patients over the study period.

## Results

### Study population

Between July 2011 and September 2023, 126 patients with scleritis were identified. After excluding 28 patients with a previously known associated systemic disease, 98 patients were included (*Supplementary Material)*. Among the overall population, 60 patients were female (61.2%), with a mean age of 45.2 ± 17.3 years, and 38 were male (38.8%), with a mean age of 47.2 ± 16.0 years. The median follow-up was 42 months [15.5–100].

**Fig. 1:**
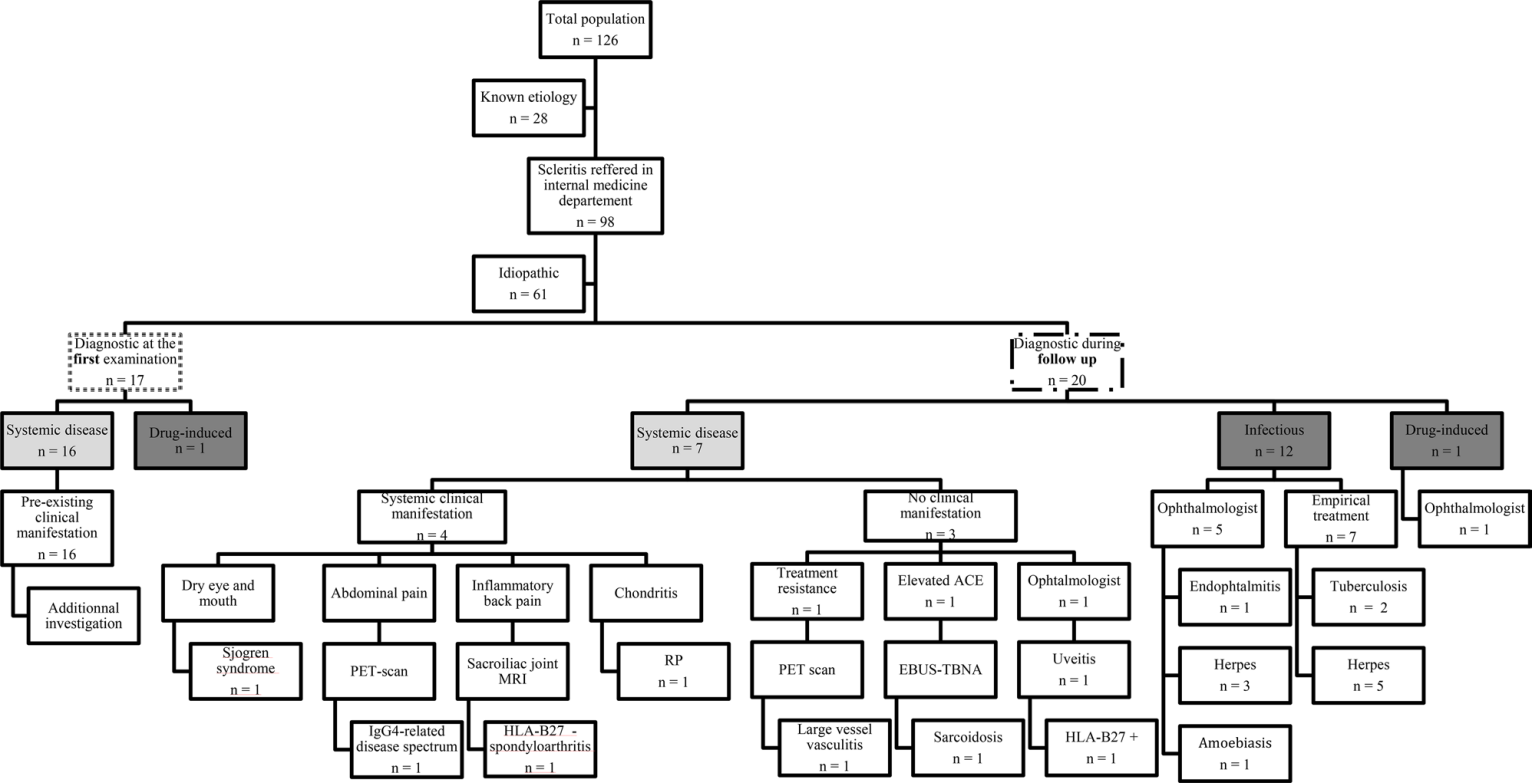
distribution of scleritis. ACE: angiotensin converting enzyme; EBUS-TBNA: endobronchial ultrasound-guided transbronchial needle aspiration; HLA: human leucocyte antigen; MRI: magnetic resonance imaging; PET scan: positron emission tomography scan; RP: relapsing polychondritis

In the internal medicine department, an associated disease was identified in 37 patients (37.8%; Table 1). The associated disease was identified during the first examination in 17 patients, whereas it was identified during follow-up in 20 patients (Fig. 1). An associated systemic disease was identified in 23 patients (23.5%): GPA (*n* = 8), sarcoidosis (*n* = 3), Behçet’s disease (*n* = 2), RP (*n* = 2), mouth and genital ulcers with inflamed cartilage (MAGIC syndrome; *n* = 1), Cogan’s syndrome (*n* = 1), large-vessel vasculitis (*n* = 1), IgG4-related disease spectrum (*n* = 1), Sjögren’s syndrome (*n* = 1), scleritis associated with HLA-B27 grouping (*n* = 1), HLA-B27-negative spondyloarthritis (*n* = 1), and systemic post-streptococcal reaction (*n* = 1). In 12 patients (12.2%), an infectious disease was diagnosed: herpetic infection (*n* = 8), tuberculosis (*n* = 2), *Staphylococcus endophthalmitis* (*n* = 1), and amoebiasis (*n* = 1). The final two patients (2.0%) had drug-induced scleritis, one in a patient treated with anti-programmed cell death-1 (pembrolizumab) for melanoma, and the other in a patient topically treated for glaucoma with a combination of brinzolamide and brimonidine tartrate.

### Ocular features at baseline and during follow-up

At presentation, 59 patients (60.2%) had diffuse anterior scleritis, 18 (18.4%) had posterior scleritis, 14 (14.3%) had nodular scleritis, 6 (6.1%) had pan-scleritis (anterior and posterior), and 1 (1.0%) had necrotising scleritis with inflammation. No significant difference in scleritis location was observed between patients with associated systemic diseases, those with infectious scleritis, and those with idiopathic scleritis (*p* = 0.213, Table 1).

In 13.3% of patients, uveitis was concomitant with the initial episode of scleritis. During follow-up, uveitis occurred in 8.2% of patients. No significant difference in the occurrence of uveitis was observed among patients with systemic disease–associated, infectious, or idiopathic scleritis (*p* = 0.225; Table 1). Scleritis was initially unilateral in most patients (*n* = 90 [91.8%]). Among patients who experienced recurrent scleritis (*n* = 80 [81.6%]), it was predominantly unilateral (*n* = 43 [53.7%]), while in 37 patients (46.3%) scleritis was alternating or bilateral. Among these 80 patients, 14 (17.5%) experienced a change in the location of scleritis compared with the initial episode (Table 2). Among these 14 patients, 6 had a systemic disease, 3 had an infectious disease (herpetic infection, tuberculosis, and amoebiasis), and in 1 patient scleritis was drug-induced.

Changes in the location of scleritis over time were more frequent among patients with associated systemic (p = *0.021*,* OR* [95% CI] *5.03 [1.27; 19.92];* Table 1). In addition, intraocular hypertension during follow-up was significantly more frequent in patients with an associated systemic disease (*p* = 0.015; OR [95% CI] 4.9, [1.37–17.54]; Table 1).

**Table 1:** Demographic, clinical, and anatomical characteristics of patients with scleritis (*n* = 98), with comparisons across the four categories

	Total population (*n* = 98)	Scleritis associated with systemic disease (*n* = 23)	Infectious scleritis (*n* = 12)	Drug-induced scleritis (*n* = 2)	Idiopathic scleritis (*n* = 61)	*p*-value^*^
Population, n (%)	98 (100.0)	23 (23.5)	12 (12.2)	2 (2.0)	61 (62.2)	
Female, n (%)	60 (61.2)	14 (60.9)	7 (58.3)	0 (0)	39 (63.9)	0.386
Mean age, years ± SD	48.0 ± 16.8	49.3 ± 14.6	53.9 ± 12.4	56.0 ± 4.2	42.9 ± 18.1	0.065
Median follow-up [IQR], months	42 [15.5–100.0]	93 [25.5–131.0]	30 [26.2–62.8]	43 [30.0–56.0]	29 [10.0–81.0]	0.126
Unilateral at the first examination, n (%)	90 (91.8)	20 (87.0)	12 (100.0)	1 (50.0)	57 (93.4)	0.123
Initial location, n (%)						0.213
Anterior						
Diffuse	59 (60.2)	15 (65.2)	7 (58.3)	0 (0)	37 (60.7)	
Nodular	14 (14.3)	2 (8.7)	3 (25.0)	1 (50.0)	8 (13.1)	
Necrotising with inflammation	1 (1.0)	1 (4.3)	0 (0.0)	0 (0)	0 (0)	
Posterior	18 (18.4)	4 (17.4)	1 (8.3)	0 (0)	13 (21.3)	
Pan-scleritis	6 (6.1)	1 (4.3)	1 (8.3)	1 (50.0)	3 (4.9)	
Multiple attacks, n (%)	80 (81.6)	21 (91.3)	11 (91.7)	2 (100.0)	46 (75.4)	0.275
Change in location of scleritis	14 (17.5)	6 (28.6)	3 (27.3)	1 (50.0)	4 (8.7)	**0.016** ^a^
Strictly unilateral during follow-up	43 (53.7)	11 (52.4)	11 (100.0)	1 (50.0)	20 (43.5)	**0.006** ^**b**^
Complications, n (%)						
IOHT	16 (16.3)	7 (28.0)	3 (25.0)	1 (50.0)	5 (8.2)	**0.019** ^**c**^
Uveitis	34 (34.7)	8 (34.8)	7 (58.3)	1 (50.0)	18 (29.5)	0.225
At the initial episode of scleritis	13 (13.3)	1 (4.4)	4 (33.3)	0 (0)	8 (13.1)	
Before scleritis	13 (13.3)	3 (13.0)	3 (25.0)	0 (0)	7 (11.5)	
After scleritis	8 (8.2)	4 (17.4)	0 (0)	1 (50.0)	3 (4.9)	
PUK	3 (3.1)	2 (8.7)	0 (0)	0 (0)	1 (1.6)	0.232

IOHT: intraocular hypertension; PUK: peripheral ulcerative keratitis; SD: standard deviation

Statistical tests:

^*^p-value = comparison across idiopathic, infectious, drug-induced, and systemic disease–associated scleritis. Categorical variables were compared using the Fisher’s exact test and continuous variables using Kruskal-Wallis test. When omnibus p-value was less than 0.05, post-hoc test for two by two multiple comparisons was performed: Dunn after Kruskal-Wallis and Marascuilo’s procedure after Fisher’s exact test

^a^*p* = 0.021 between systemic disease and idiopathic scleritis. No difference between infectious disease and idiopathic scleritis. No difference between systemic disease and infectious disease

^b^*p* = 0.002 between infectious disease and idiopathic scleritis, *p* = 0.007 between infectious disease and systemic disease. No difference between systemic disease and idiopathic scleritis. The odds ratio could not be estimated because the event occurred in 100% of cases within one comparison group

^c^*p* = 0.015 between systemic disease and idiopathic scleritis. No difference between infectious disease and idiopathic scleritis. No difference between infectious disease and systemic disease

**Table 2:** Clinical features of recurrent scleritis (80 patients)

Overall population, *n*	98
Recurrent scleritis, n	80
No change, n, (%)	66 (82.5)
Change in scleritis, n (%)	14 (17.5)
Diffuse to nodular	1
Diffuse to pan scleritis	1
Diffuse to posterior	5
Nodular to necrosing	1
Nodular to diffuse	1
Nodular to posterior	1
Posterior to diffuse	1
Posterior to pan scleritis	2
Pan scleritis to diffuse scleritis	1

### Clinical features suggestive of an associated systemic disease present prior to the initial examination

Systemic manifestations preceded the first episode of scleritis in 16 patients (16.3%) and were subsequently confirmed at the time of systemic examination, aiding in the identification of an associated systemic disease.

General health deterioration was noted in 8 patients, 4 of whom had GPA. ENT manifestations such as haemorrhagic rhinorrhoea led to the identification of GPA in 7 patients, while sensorineural hearing loss and dizziness led to the identification of Cogan’s syndrome. In addition, in 2 patients, the presence of chondritis suggested RP (*n* = 1) or MAGIC syndrome (*n* = 1). A history of tonsillitis preceding the onset of scleritis led to the identification of a post-streptococcal aetiology. Regarding dermatological examination, it led to the identification of 1 GPA (gravity-independent leg ulcers located above the malleoli, in a patient without ENT manifestations), 2 Behçet’s disease (pseudofolliculitis, aphthosis), 1 MAGIC syndrome (pseudofolliculitis, aphthosis), and 1 sarcoidosis (erythema nodosum).

Inflammatory joint pain contributed to the identification of the underlying disease in three patients, which were Behçet’s disease, sarcoidosis, and post-infectious scleritis with reactive arthritis (Table 3). Of note, several clinical manifestations could occur simultaneously in the same patient.

**Table 3:** Clinical features associated with identified systemic diseases

	Digestive	Dermatological examination	ENT	Joint	General health deterioration	No clinical sign
Systemic disease (*n* = 23)	1	6	12	4	8	3
GPA (*n* = 8)		1	7		4	
Sarcoidosis (*n* = 3)		1		1	1	1
Behçet’s disease (*n* = 2)		2		1	1	
Sjögren syndrome (*n* = 1)		1				
IgG4-related disease spectrum (*n* = 1)	1					
HLA-B27 negative spondyloarthropathy (*n* = 1)				1		
Large-vessel vasculitis (*n* = 1)						1
Cogan’s syndrome (*n* = 1)			1			
MAGIC (*n* = 1) and RP (*n* = 2)		1	3		2	
Scleritis HLA-B27 ^+^ (*n* = 1)						1
Post streptococcal reaction (*n* = 1)			1	1		

ENT: ear, nose and throat; GPA: granulomatosis with polyangiitis; MAGIC: mouth and genital ulcers with inflamed cartilage; RP: relapsing polychondritis

### Contribution of follow-up to the identification of associated systemic diseases

Among the 23 patients with an associated systemic disease, scleritis represented the initial manifestation in 7 cases (30.4%) (Fig. 1). In 4 patients (17.4%), the systemic examination became positive during follow-up, enabling identification of the underlying systemic disease.

One patient presented with dry mouth and eyes 12 years after the first episode of scleritis, which led to the identification of Sjögren’s syndrome. In another patient, IgG4-related disease spectrum was identified nine years after the first episode of scleritis, following the onset of abdominal pain.

A third patient presented with low back pain and MRI-confirmed sacroiliitis, occurring three years after the first episode of scleritis, which led to the identification of HLA-B27-negative spondyloarthritis. In the last patient, the onset of chondritis which occurred two years after scleritis initially associated with aortitis, led to the identification of RP (Fig. 1).

In the remaining 3 patients (13.0%), systemic examination was unremarkable, and the associated systemic disease was identified through additional investigations: a large-vessel vasculitis was detected on a PET scan performed because of treatment-resistant scleritis; sarcoidosis was identified through elevated ACE levels and lymphadenopathy on thoracic CT, confirmed by endobronchial ultrasound-guided transbronchial needle aspiration (EBUS-TBNA); and finally, HLA-B27–associated scleritis was recognised following the onset of a typical HLA-B27–related uveitis within a month after the initial episode of scleritis.

Of note, repeated examinations helped identify infectious scleritis in 5 of the 12 patients (41.7%). Three cases of herpetic scleritis were identified, all exhibiting corneal anaesthesia and kerato-uveitis; one case was confirmed by an aqueous humour tap.

The secondary occurrence of keratoneuritis suggested an amoebic infection, which was confirmed by corneal scraping that identified the pathogen. Finally, a positive culture for *Staphylococcus epidermidis* in the aqueous humour tap led to the identification of endophthalmitis. Additionally, the empirical treatment initiated during the evaluation with the internist — comprising valaciclovir for herpetic scleritis (*n* = 5) and antitubercular drugs for tuberculous scleritis (*n* = 2) — supported these identifications. Regarding drug-induced scleritis, ophthalmological examination during follow-up led to the identification of cases related to brinzolamide–brimonidine tartrate, which was subsequently discontinued.

### Contribution of additional investigations

#### Contribution of imaging

Among the overall population, 89 patients (90.8%) underwent thoracic imaging: 53 had chest X-rays, 62 had CT scans, and 26 underwent both modalities. Abnormal findings were observed in 15 patients (12 CT scans; 3 X-rays confirmed by CT scans), leading to the identification of a systemic disease in 5 cases: sarcoidosis with lymphadenopathy (*n* = 3), GPA due to the identification of an intra-alveolar haemorrhage (*n* = 1), and RP due to the identification of aortitis (*n* = 1). Sinus CT scans were performed in 22 patients, including 3 without ENT manifestations. This contributed to the identification of the underlying disease in 6 cases (5 GPA and 1 RP): in the patient with RP, the disease was identified through the presence of cartilage deformities; in patients with GPA, it was identified through sinusitis (*n* = 4) and nasal septum perforation (*n* = 1). Finally, 12 patients underwent a PET scan, which contributed to identifying the underlying disease in 6 cases: IgG4-related disease spectrum, large-vessel vasculitis, sarcoidosis, Cogan’s syndrome, MAGIC syndrome, and RP.

#### Contribution of laboratory tests

ANCA tests were performed in 96 of the 98 patients (98.0%); 21 had positive ANCA, and 8 of these patients had a positive systemic examination. Among the 21 patients, 14 had ANCA positivity without ELISA specificity; 6 of them had associated diseases: sarcoidosis, Cogan’s syndrome, herpetic scleritis, large-vessel vasculitis, HLA-B27-negative spondyloarthritis, and IgG4-related disease spectrum. The 8 remaining patients had not developed any systemic disease after the median [IQR] follow-up of 33 [1–176] months.

Seven patients had ANCA positivity with ELISA specificity (6 anti-PR3 and 1 anti-MPO), leading to the identification of 6 cases of ANCA-associated vasculitis. In the last patient, who had anti-PR3 at a low titre (3.7, *N* < 3), no underlying systemic disease was identified after 140 months of follow-up.

The presence of ANCA detected on IF at the initial examination was associated with GPA (OR [95% CI] 14.6 [2.7; 79.4]) in patients with positive ANCA compared to those with negative ANCA (*p* = 0.001; Table 4).

Similarly, ANCA positivity with specificity was significantly associated with GPA (OR [95% CI] 261 [20.6; 3306.3]) compared to ANCA negativity (*p* < 0.001;Table 4). The positive predictive value (PPV) of ANCA without specificity was 29%, and the negative predictive value (NPV) was 97%. For ANCA with specificity, the PPV was 86% (Table 5). Of note, ANCA tests were repeated in 32 of the 61 patients with idiopathic scleritis, after a median [IQR] 22 months [8.7–51.0] following the initial test. No repeated assay was positive.

A total of 89 of the 98 (90.9%) patients were tested for RF and anti-CCP and 3 patients had positive RF and anti-CCP. One patient had RA, but the scleritis was associated with an endophthalmitis.

In the second patient, herpetic scleritis was identified based on the presence of uveitis, corneal anaesthesia, a favourable response to valaciclovir, and the absence of joint manifestations after 26 months of follow-up. The third patient had no systemic manifestations, and the systemic examination and imaging remained unremarkable for RA after 60 months of follow-up. Repeated RF and anti-CCP tests were performed in 29 patients after a median [IQR] 21 months [7.0–47.0] following the initial test. None of the patients developed positive RF or anti-CCP.

Among the overall population, 68 (69.4%) had an ACE assay (normal < 70) performed. The assays were positive in 8 patients, including 3 with markedly elevated levels (ACE > 90), which were associated with sarcoidosis.

In 2 patients, sarcoidosis was confirmed by histopathology, which revealed the presence of granulomas with giant cells without caseous necrosis (bronchial spur biopsy and EBUS-TBNA). In the last case, a history of erythema nodosum and lymphadenopathy in Barrety’s space suggested the condition, although no histological confirmation was available.

**Table 4:** ANCA and the risk of developing granulomatosis with polyangiitis

	GPA + *n* = 8	GPA – *n* = 88	OR [95% CI]	*p*-value
ANCA positive without specificity	6	15	14.6 [2.7; 79.4]	0.001
ANCA positive with specificity	6	1	261.0 [20.6; 3306.3]	< 0.001
Extra-articular signs (ENT, dermatological)	8	29	35.5 [2.0; 635.0]	< 0.001

ANCA: anti-neutrophil cytoplasmic antibodies; CI: confidence interval; ENT: ear, nose, and throat; GPA: granulomatosis with polyangiitis; OR: odds ratios

﻿Odds ratios (OR) with 95% confidence intervals (CI) were calculated using univariate logistic regression. Values represent counts of patients with or without GPA for each feature. Some features overlap, therefore totals exceed the number of GPA cases. p-values < 0.05 were considered statistically significant

**Table 5:** Diagnostic performance of ANCA testing in patients with scleritis without an identified associated systemic disease

	LR+	LR-	Sensitivity	Specificity	PPV	NPV
ANCA without specificity	4.41	0.30	0.75	0.83	0.29	0.97
ANCA with specificity	68.2	0.25	0.75	0.99	0.86	0.98

ANCA: anti-neutrophil cytoplasmic antibodies; LR-: likelihood ratio for a negative test; LR+: likelihood ratio for a positive test; NPV: negative predictive value; PPV: positive predictive value

## Discussion

This study highlights the importance of a systematic evaluation combining systemic examination and additional investigations in identifying associated diseases in patients with scleritis of initially unknown origin. Our findings suggest that such an approach can reveal an associated disease in approximately 40% of patients – most often systemic – either at baseline or during follow-up. While these results support the usefulness of a structured systemic assessment, they remain observational and should not be interpreted as proving causality.

In 20% of patients with scleritis referred to the internal medicine department for systemic evaluation, a positive systemic examination played a key role in identifying an associated systemic disease. These results support the value of structured systemic assessment, and prospective studies will be needed to validate this approach.

Systemic manifestations were present before the first episode of scleritis in approximately three-quarters of patients, while in one-quarter a subsequent systemic examination revealed new manifestations that contributed to the identification of an associated systemic disease. This sequential diagnostic process underscores that many systemic diseases associated with scleritis evolve over time and that a single baseline evaluation may be insufficient, thereby highlighting the value of longitudinal follow-up.

In addition, the present study suggests the importance of an interview focused on ENT manifestations and a dermatological examination. It also highlights the value of testing for ANCA and determining its specificity in the initial assessment of scleritis. However, our data indicate that repeating these tests without new clinical manifestations has limited diagnostic yield.

The characteristics of the present population were similar to those reported in the main series in the literature (Table 6) regarding sex ratio and the predominantly anterior location of scleritis [3, 4, 12, 33–38]. Herein, a younger age at presentation and a higher number of recurrences were observed [33]. These differences may reflect a referral bias to internal medicine rather than true epidemiological variation. The present study found that change in the location of scleritis was uncommon, although more frequent than in other series [33, 34, 38] with similar follow-up (Table 6). This change in location was significantly associated with the presence of a systemic a finding that was not emphasised in other series [3, 4, 15, 39]. However, given our sample size, this result should be interpreted with caution because of limited statistical power.

The proportion of associated systemic diseases in patients with scleritis initially considered of unknown origin was higher in our study compared to that reported by Sainz de la Maza et al. (9.6%), Wieringa et al. (7.7%) [4, 37].

This difference is likely explained by the systematic examination of patients performed by internists in the present study, improving the identification of subtle or early systemic signs rather than indicating a true difference in disease prevalence. Therefore, our findings reinforce — but do not establish — the value of multidisciplinary evaluation in complex scleritis.

Half of the associated diseases were identified during follow-up. These diagnoses arose through four mechanisms: emergence of new systemic manifestations, new laboratory or imaging findings, new ophthalmologic examinations, or response to empirical treatment. This sequential diagnostic process underscores that many systemic diseases associated with scleritis evolve over time and that a single baseline evaluation may be insufficient.

An associated systemic disease was identified at the initial examination in the majority of patients, based on pre-existing systemic manifestations. In others, it was diagnosed later, following the appearance of new systemic manifestations, up to 12 years after the first episode. In nearly 15% of patients, additional imaging or ophthalmologic examinations contributed to the identification of the systemic disease.

Conversely, in infectious diseases, no pre-existing systemic manifestations were found. In approximately 40% of cases, diagnosis relied mainly on ocular course and therapeutic response, highlighting the difficulty of distinguishing infectious from immune-mediated scleritis based solely on baseline evaluation.

Scleritis is a frequent ophthalmic manifestation of GPA (15–30%) [40–42]. Although scleritis can be the first manifestation of GPA, it is only found in a minority of patients [42, 43]; scleritis was present at the time of GPA in only 5.7% of the 876 patients in the series published by Junek et al. [44], without knowing the chronology of clinical manifestation. Herein, the scleritis observed in patients with GPA was often preceded by other clinical manifestations. Therefore, a systemic examination performed by an internist could play a key role in the early identification of GPA through the detection of prior clinical manifestations, particularly those involving ENT or dermatological findings.

This study confirmed the importance of ANCA specificity [12, 45]. However, the sensitivity of this biomarker is not perfect, and GPA should still be considered when ANCA is absent. This was the case in two patients herein as well as in two patients in a previous study [3]. The present study also suggested that repeating ANCA testing is not useful in the absence of systemic clinical manifestations suggestive of GPA. These findings support current practice but remain exploratory, given the limited sample size.

In the present study, no scleritis was the initial manifestation of RA, whereas it was the case in 9.2% of patients in Lin et al.‘s series and 4.2% in Abd El Klatif et al.‘s series [12, 46]. This discrepancy could be explained by a different referral of patients. Patients with joint manifestations and a positive RF and/or anti-CCP could be preferentially referred to a rheumatologist rather than an internist, which may have reduced the proportion of RA. Moreover, no case of idiopathic scleritis progressing to RA was observed in the presence of positive RF or anti-CCP. This contrasts with the findings of Lin et al.‘s series [12], in which scleritis that was initially considered idiopathic was associated with a fifty-time greater risk of RA in patients with positive RF. In this previous study, 5 patients developed RA despite the absence of initial inflammatory arthralgia when RF was positive, which was not found herein; as found with ANCA, neither RF nor anti-CCP became positive during follow-up.

Although sarcoidosis was observed in a few patients herein, the proportion was greater than in other series in the literature; of note, in the series by Sainz de la Maza et al. no sarcoidosis was diagnosed among the 500 patients [4, 35, 36]. The prevalence of scleritis in sarcoidosis is estimated to be lower than 3% [47]. Although our data suggest the usefulness of ACE testing and thoracic CT for scleritis without identified associated systemic disease, this remains a hypothesis that requires confirmation in larger cohorts.

The frequency of RP associated with scleritis in the present study was within the range reported in the literature, from 0.87 to 7.5% [34, 36, 38]. Consequently, evaluation for chondritis should be systematic in patients with scleritis, as it represents the most frequent ophthalmic manifestation of RP (26–47%) [48–50]. Although our findings support systematic assessment for chondritis, they remain descriptive and are not sufficient to justify universal screening. In the absence of clinical manifestations suggesting an underlying cause, additional investigations enabled the identification of an associated disease. Taken together, these findings raise hypotheses regarding underlying mechanisms. The strong contribution of systemic manifestations preceding scleritis supports the concept that ocular inflammation often represents a late or secondary expression of systemic inflammatory or autoimmune disorders.

There is no consensus regarding the evaluation of systemic associations in scleritis. However, based on the present results and on the literature [3, 12, 13], it seems reasonable to propose the following initial assessment: a complete blood count, CRP, creatinine measurement, serologic syphilis testing, ANCA with determination of their specificity, RF and anti-CCP antibodies, followed by ACE and chest imaging. Although no cases of syphilis were detected, screening remains necessary because of the potentially harmful consequences. Furthermore, in the cohort studied by *Cristina Arruza et al.*., syphilis emerged as the most frequent infectious cause of scleritis [51].

Due to the low prevalence of tuberculous scleritis, the QuantiFERON^®^-TB Gold In-Tube test should be performed in cases of known tuberculosis exposure, when scleritis is resistant to first-line treatment, or for pre-therapeutic assessment [52]. However, our study does not establish this as a validated diagnostic strategy, and prospective evaluation is required.

This study has several limitations that must be acknowledged. First, its retrospective design introduces inherent constraints, including missing data, heterogeneity in documentation, and the absence of standardised and uniform evaluation across all patients. In addition, the duration of follow-up varied between individuals and may not have been sufficient to identify systemic diseases that may emerge over time, such as rheumatoid arthritis, which can be diagnosed up to two years after an initial episode of idiopathic scleritis with positive RF [12]. Second, the sample size remains limited, which reduces statistical power and limits the robustness analyses. Third, potential selection bias must be considered. Because patients were recruited through internal medicine departments rather than ophthalmology department —as is typically the case in other studies—our cohort may overrepresent more severe or complex cases of scleritis, thereby overestimating the proportion of associated systemic diseases. The absence of external validation further restricts the generalisability of our findings. Another limitation is the potential misclassification of patients. This is particularly relevant for infectious aetiologies such as herpetic scleritis, which is often diagnosed clinically without confirmatory biopsy or aqueous tap [7]. Moreover, the handling of missing data and the lack of a formal assessment of inter-observer variability—both in ophthalmologic and systemic evaluations—may have influenced diagnostic classification. These factors may lead to an overestimation of the prevalence of systemic disease and limit generalisability. Finally, diagnostic criteria for several systemic diseases evolved during the study period, introducing temporal heterogeneity that may have affected case definition.

Despite these limitations, this study provides valuable insight into the diagnostic pathways of scleritis and helps identify priorities for future research. Prospective, multicentre studies using standardised diagnostic strategies and predefined follow-up schedules will be essential to validate these findings. In this regard, the development of a structured diagnostic approach—similar to the ULISSE protocol designed for uveitis (ULISSE; Uveitis: clinical and medico-economic evaluation of a standardised strategy for the etiological diagnosis) [53]—could serve as a relevant model for future prospective evaluation in scleritis. In this standardised strategy, all scleritis patients could be referred to internal medicine, in which they would undergo standardised clinical questioning and first-, second-, and third-line investigations.

In conclusion, our results support the importance of a systematic and longitudinal approach for identifying associated systemic diseases in patients with scleritis. While they reinforce existing evidence, several observations remain exploratory and require confirmation in standardised prospective cohorts.

**Table 6:** Comparative characteristics of major published scleritis cohorts and the present study

	Tuft23/11/2025 22:17:00 1991	Jabs[34] 2000	Akpek[3] 2004	Lin[12] 2008	Raiji[35] 2009	Erkanli[36] 2010	Sainz De La Maza [4] 2012	Wieringa[37] 2013	Bernauer[38] 2014	Thong[1] 2020	Present study
n	290	92	243	119	86	114	500	104	40	58	98
Age (years, mean or median)	51.5	51.0	52.0	49.4	49.5	48.0	53.7	51.5	51.0	46.0	48.0
Follow-up (in months)	82.8 [8–270]	NA	20.4 [0–199]	8.0 [0–72]	30.3	16.2 [1–56]	20.4 [0–60]	38.2 [3–154]	NA	23.0 [2.4–68.8]	42.0 [15.5–100]
Female (n, %)	NA	69 (71.1)	170 (70.0)	82 (68.9)	61 (70.9)	82 (71.9)	355 (71.0)	63 (60.6)	26 (65.0)	41 (71.0)	60 (61.2)
Unilateral (n, %)	216 (74.5)	48 (49.5)	123 (50.6)	81 (68.0)	NA	80 (70.2)	294 (58.8)	64 (61.5)	26 (62.0)	44 (76.0)	43 (53.7)
Anterior (n, %)	123 (42.0)	58 (60.0)	161 (66.4)	55 (46.2)	11 (12.8)	47 (42.0)	375 (75.0)	36 (34.6)	NA	34 (59.0)	59 (60.2)
Posterior (n, %)	35 (12.0)	7 (7.2)	20 (8.4)	16 (13.4)	7 (8.1)	11 (9.0)	31 (6.0)	4 (3.8)	6 (15.0)	8 (14.0)	18 (18.4)
Nodular (n, %)	90 (31.0)	20 (20.6)	41 (16.8)	36 (30.3)	48 (55.8)	49 (43.0)	71 (14.2)	20 (19.2)	NA	16 (28.0)	14 (14.3)
NWI (n, %)	42 (15.0)	12 (12.3)	17 (7.6)	13 (10.9)	10 (11.6)	7 (6.0)	20 (4.0)	6 (5.8)	3 (7.5)	0 (0)	1 (1.0)
NWTI (n, %)	NA	0	2 (0.8)	2 (1.7)	0 (0)	0 (0)	3 (0.6)	9 (8.7)	2 (5.0)	0 (0)	0 (0)
Change in type (n, %)	38 (12)	6 (6.1)	NA	NA	NA	11 (9.6)	NA	NA	4 (10.0)	NA	14 (17.5)
PUK (n, %)	NA	17 (17.5)	15 (6.2)	NA	NA	6 (5.0)	37 (7.4)	19 (18.3)	3 (7.5)	3 (4)	3 (3.1)
Uveitis (n, %)	NA	24 (24.7)	72 (29.8)	14 (11.8)	51 (59.3)	16 (14.0)	132 (26.4)	47 (45.6)	NA	12 (17.0)	34 (34.7)
SD after scleritis (n, %)	NA	NA	20 (8.2)	25 (27.5)	5 (5.8)	13 (11.0)	48 (9.6)	8 (7.7)	3 (7.5)	9 (15.0)	23 (23.5)
Infectious (n, %)	NA	18 (18.7)	17 (7.0)	5 (4.2)	4 (4.7)	1 (0.9)	48 (9.6)	3 (2.9)	NA	1 (2)	12 (12.2)
RA (n, %)	30 (10.3)	17 (17.5)	37 (15.2)	22 (18.5)	10 (11.6)	8 (7.0)	32 (6.4)	14 (13.5)	6 (15.0)	11 (19.0)	0 (0)
GPA (n, %)	11 (3.8)	7 (7.2)	22 (9.1)	8 (6.7)	1 (1.2)	6 (5.2)	14 (2.8)	7 (6.7)	3 (7.5)	0 (0)	8 (8.2)
RP (n, %)	6 (2.1)	3 (3.1)	4 (1.6)	3 (2.5)	0 (0)	1 (0.9)	11 (2.2)	2 (1.9)	3 (7.5)	2 (3.0)	2 (2.0)
Sarcoidosis (n, %)	NA	0 (0)	NA	2 (1.7)	2 (2.3)	3 (3.0)	0 (0)	0 (0)	0 (0)	1 (2)	3 (3.1)
Internist examination	NA	If suspected SD	If suspected SD	If suspected SD	NA	If suspected SD	If suspected SD	If suspected SD	NA	NA	Systematically

GPA: granulomatosis with polyangiitis; NA: not available; NWI: necrosing with inflammation; NWTI: necrosing without inflammation; PUK: peripheral ulcerative keratitis; RA: rheumatoid arthritis; RP: relapsing polychondritis; SD: systemic disease

## Conclusion

The present study highlights the importance of the initial systemic examination and follow-up. These assessments contributed to identifying associated systemic diseases in one-fifth of patients with scleritis who had no previously recognised systemic involvement. In most cases, systemic manifestations preceded the first episode of scleritis. The utility of repeating laboratory tests in the absence of new clinical manifestations in idiopathic scleritis appears limited. However, prospective studies assessing standardised strategies for the identification of associated systemic diseases in scleritis are warranted.

## Supplementary Information

Below is the link to the electronic supplementary material.

Supplementary Material 1

Supplementary Material 2

Supplementary Material 3

## Data Availability

The datasets used and/or analysed during the current study are available from the corresponding author on reasonable request.

## Abbreviations

ACR: American College of Rheumatology
ACE: Angiotensin-converting enzyme
ANA: Anti-nuclear antibodies
ANCA: Anti-neutrophil cytoplasmic antibodies
Anti-CCP: Anti-cyclic citrullinated peptide
ASAS: Assessment of SpondyloArthritis International Society
CBC: Complete blood count
CRP: C-reactive protein
CT: Computed tomography
ELISA: Enzyme-linked immunosorbent assay
ENT: Ear, nose, and throat
EULAR: European Alliance of Associations for Rheumatology
EBUS-TBNA: Endobronchial ultrasound-guided transbronchial needle aspiration
GPA: Granulomatosis with polyangiitis
HLA: Human leukocyte antigen
IQR: Interquartile range
IWOS: International Workshop on Sarcoidosis
MAGIC syndrome: Mouth and genital ulcers with inflamed cartilage
MRI: Magnetic resonance imaging
NPV: Negative predictive value
OR: Odd ratio
PET scan: Positron emission tomography scan
PPV: Positive predictive value
RA: Rheumatoid arthritis
RF: Rheumatoid factor
RP: Relapsing polychondritis
SD: Standard deviation
SLE: Systemic lupus erythematosus
SPE: Serum protein electrophoresis
ULISSE: Uveitis: Clinical and medico-economic evaluation of a standardised strategy for the etiological diagnosis
