# Supplementary material for: Scleritis and associated systemic diseases: contribution of systemic examination, follow-up, and additional investigations
Source: J Ophthalmic Inflamm Infect. 2025 Dec 25;16:7. doi: 10.1186/s12348-025-00566-7 (PMC12847570; doi:10.1186/s12348-025-00566-7)
Supplement: Supplementary file 1 — Supplementary Material 1 [file 12348_2025_566_MOESM1_ESM.docx]

*Supplemental material*

|  | Known etiology  n = 28 |
| --- | --- |
| Female (n, %) | 15 (53.6) |
| Age (years, mean) | 54.9 |
| Unilateral at the first examination (n, %) | 26 (92.9) |
| Multiple attacks (n, %) | 18 (64.3) |
| Strictly unilateral during follow-up (n, %) | 13 (72.2) |
| Initial location |  |
| Anterior (n, %) | 17 (60.7) |
| Nodular (n, %) | 3 (10.7) |
| Necrotizing without inflammation (n, %) | 1 (3.6) |
| Necrotizing with inflammation (n, %) | 1 (3.6) |
| Posterior (n, %) | 3 (10.7) |
| Pan-scleritis (n, %) | 3 (10.7) |
| Complications |  |
| IOHT (n, %) | 4 (14.3) |
| Uveitis (n, %) |  |
| At the diagnostic of scleritis (n, %) | 1 (3.6) |
| Before scleritis (n, %) | 3 (10.7) |
| After scleritis (n, %) | 2 (7.1) |
| PUK (n, %) | 4 (14.3) |
| Etiology (n, %) | 28 |
| GPA (n, %) | 5 (17.9) |
| RA (n, %) | 5 (17.9) |
| Inflammatory bowel diseases | 5 (17.9) |
| SLE | 2 (7.1) |
| Berger’s disease | 2 (7.1) |
| Sarcoidosis | 2 (7.1) |
| Others* | 7 (25.0) |

*GPA: granulomatosis with polyangiitis; IOHT: intraocular hypertension; PUK: peripheral ulcerative keratitis RA: rheumatoid arthritis, SLE: systemic lupus erythematosus*

**: microscopic polyangiitis; scleritis associated with HLA-B27, spondyloarthritis; celiac disease; Wiskott-Aldrich syndrome; vacuoles, E1-Enzyme, X-linked, Autoinflammatory, Somatic-disease (VEXAS); and a poorly identified systemic disease secondary to low-grade B-cell lymphoid disorder.*
