# Supplementary material for: Scleritis and associated systemic diseases: contribution of systemic examination, follow-up, and additional investigations
Source: J Ophthalmic Inflamm Infect. 2025 Dec 25;16:7. doi: 10.1186/s12348-025-00566-7 (PMC12847570; doi:10.1186/s12348-025-00566-7)
Supplement: Supplementary file 2 — Supplementary Material 2 [file 12348_2025_566_MOESM2_ESM.docx]

# Supplementary table : Imaging Devices, Acquisition Protocols, and Calibration Procedures

| Imaging modality | Device model(s) & site | Acquisition protocol | Calibration / Quality Control procedure |
| --- | --- | --- | --- |
| Chest X‑ray | Standard digital radiography units (Croix‑Rousse & Edouard Herriot) | PA and lateral views; exposure adapted to patient morphology | Daily detector calibration; weekly quality‑control checks |
| Chest CT‑scan | • Edouard Herriot: GE Revolution GSI • Croix‑Rousse: Philips Spectral CT 7500 | Helical acquisition; 0.625–1.25 mm slices; with/without contrast | Automatic tube current modulation; daily phantom calibration |
| Sinus CT‑scan | Same CT platforms as above | High‑resolution sinus protocol; 0.6–1 mm slices; bone algorithm | Same calibration as chest CT |
| Osteoarticular X‑ray | Digital radiography units (Croix‑Rousse) | Standard AP/lateral views | Daily detector calibration; weekly QC |
| MRI (osteoarticular imaging) | 1.5 T and 3 T MRI systems (Edouard Herriot) | T1, T2, STIR; fat-suppression; 3–4 mm slices | Daily system calibration; weekly phantom QC |
| PET‑scan | Integrated PET‑CT systems (HCL network) | Whole‑body, attenuation‑corrected, 18F‑FDG protocol | Daily normalization; uniform‑phantom calibration |
